# Increased Risk of Liver Cirrhosis during Azathioprine Therapy for Crohn's Disease

**DOI:** 10.1155/2020/6726384

**Published:** 2020-01-28

**Authors:** Jenny Roselli, Tommaso Innocenti, Erica Nicola Lynch, Laura Parisio, Giuseppe Macrì, Monica Milla, Tommaso Mello, Andrea Galli, Stefano Milani, Mirko Tarocchi

**Affiliations:** Department of Experimental and Clinical Biomedical Sciences “Mario Serio”, University of Florence, Florence, Italy

## Abstract

Azathioprine is a cornerstone of the therapy of Crohn's disease. Unfortunately, infections and malignancies are relatively common adverse effects related to this drug; however, cirrhosis is exceptionally reported as a side effect. We report the case of a 49-year-old male patient with ileocolonic steno-penetrating Crohn's disease who developed hepatic cirrhosis while treated with azathioprine. After taking azathioprine for 3 years with regular follow-up, he developed pancytopenia, and liver cirrhosis was diagnosed with ultrasound, abdomen computed tomography scan, transient elastography, and liver biopsy. As all other causes of liver damage were excluded, azathioprine was believed to be the cause of liver injury and therefore was interrupted.

## 1. Introduction

Thiopurines (azathioprine AZA and 6-mercaptopurine 6-MP) are the gold standard for the treatment of steroid-dependent ileocolonic Crohn's disease with moderate-to-severe disease activity [1].

There are numerous adverse events related to these drugs, such as nonmelanoma skin cancer (NMSC) [2–5], lymphoma, infections, and pancreatic and liver injury [6]. In most cases (5–15% of all patients on thiopurine therapy [6]), elevated liver enzymes (glutamate oxaloacetate transaminase (GOT) and glutamate pyruvate transaminase (GPT)) detect the presence of liver damage, although patients generally remain asymptomatic. The liver function test usually normalizes after reducing or stopping treatment [3, 6, 7]. Less commonly, thiopurines can cause cholestatic hepatitis, and in rare cases, they can be associated with noncirrhotic portal hypertension, caused by nodular regenerative hyperplasia or by hepatic veno-occlusive disease [3, 6, 8].

In this report, we present the case of a patient with Crohn's disease who developed hepatic cirrhosis while treated with AZA.

## 2. Case Report

**Figure 1:**
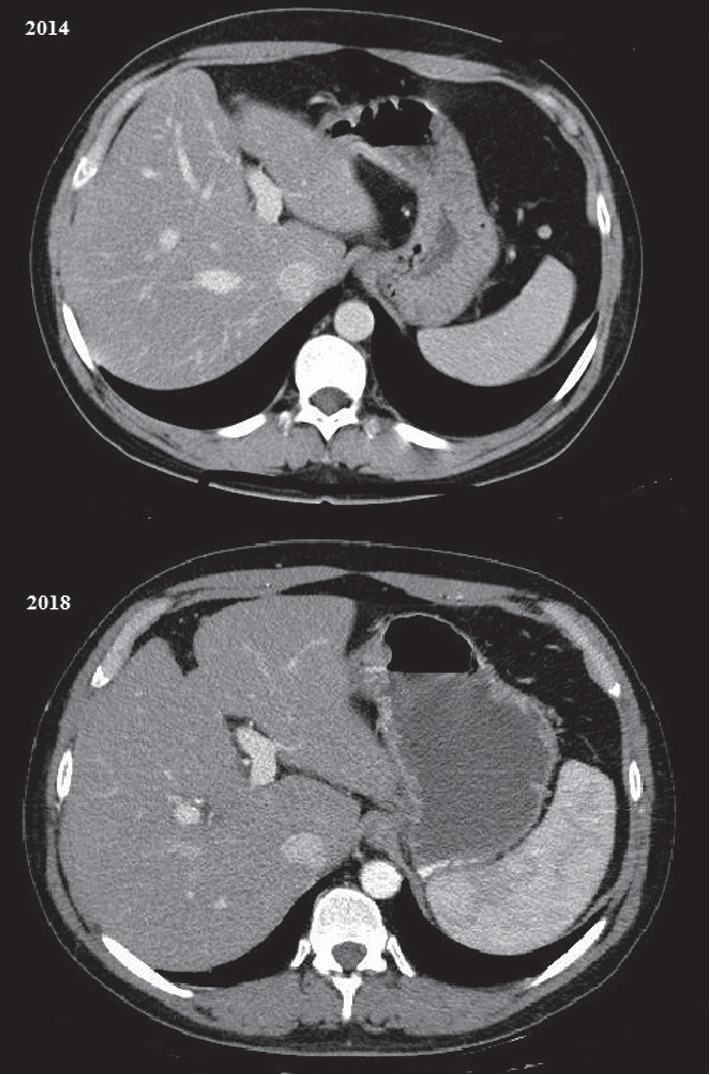
Comparison between arterial-phase abdomen CT scans from 2014 (before AZA therapy) and 2018 (after AZA therapy).

**Figure 2:**
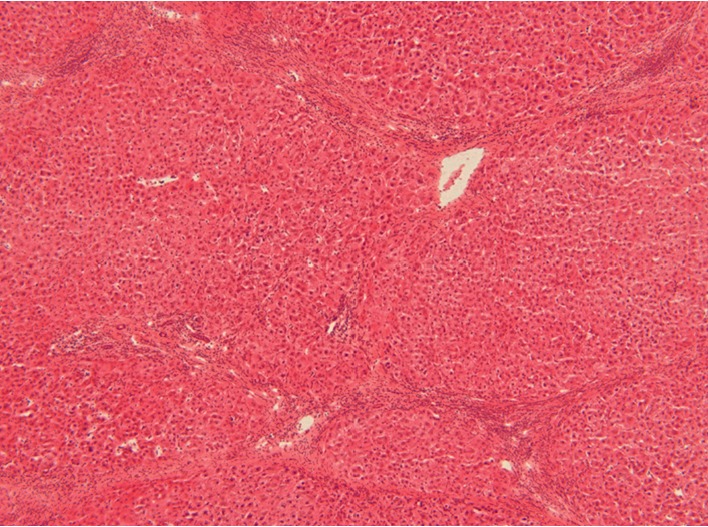
Findings of histology: marked bridging with regenerative nodules; hematoxylin and eosin stain.

A 49-year-old male Italian patient was diagnosed with ileocolonic steno-penetrating Crohn's disease in 1997. He was initially treated with oral steroids and achieved disease remission. He was then treated with mesalazine alone for many years. The patient was referred to our center in September 2014 for a disease relapse. He underwent a colonoscopy and a digestive MRI that showed wall thickening of the last 35 cm of the ileum and of the descending colon; thus, we proposed a step-up therapy with azathioprine 2 mg/kg/day, which the patient started in October 2014. The patient regularly attended follow-up visits every three months, and routine blood tests revealed normal complete blood count, C-reactive protein, and pancreatic and hepatic enzymes until December 2017, when they gave the following results: pancytopenia (WBC 2200 cells/mm^3^, RBC 2500 cells/mm^3^, and PLT 108000 cells/mm^3^), high levels of bilirubin (total 2.2 mg/dL and direct 0.77 mg/dL), and normal levels of GOT, GPT, and *γ*GT (respectively, 41, 26, and 59 U/L). The patient was diagnosed with liver cirrhosis with ultrasound, abdomen computed tomography scan, transient elastography, and a liver biopsy. Ultrasound showed signs of liver cirrhosis, like hypertrophy of the left lobe of the liver, ragged edges, and slightly increased liver echogenicity; portal vein Doppler ultrasound showed a portal diameter of 1 cm, hepatopetal portal venous circulation with a venal flow of 16 cm/sec, thin suprahepatic veins, and splenic enlargement (17 cm). The abdomen CT scan is shown in Figure 1. Transient elastography results were as follows: stiffness 21.6 kPa, IQR 3.4 kPa, and controlled attenuation parameter (CAP) 246 dB/m. The liver biopsy (Figure 2) showed signs of liver cirrhosis, excluding nodular regenerative hyperplasia, veno-occlusive hepatic disease, or a biliary tract disease. The gastroscopy showed congestive gastropathy. Research of possible viral liver damage (HAV, HBV, HCV, HEV, EBV, and CMV) came out negative. There were no laboratory signs of dyslipidemia, insulin resistance, or diabetes mellitus. Laboratory tests for autoimmune liver diseases (research of antinuclear antibodies (*ANA*), antineutrophil cytoplasmic antibodies (*ANCA*), anti-soluble liver antigen antibodies (*anti-SLA*), anti-smooth muscle antibodies (*ASMA*), anti-SP100 antibodies, anti-liver kidney microsome antibodies (*anti-LKM*), antimitochondrial antibodies (*AMA*), anti-mitochondrial M2 antibodies (*AMA-M2*), anti-GP210 antibodies, and anti-liver cytosol type 1 antibodies (*anti-LC1*)) were all negative. Specific tests were performed to exclude iron and copper overload. As all other causes of liver damage were excluded, AZA therapy was believed to be the cause of liver injury and therefore was interrupted. Six months after discontinuation of AZA treatment, ultrasound and CT scans revealed similar results, whereas white cells and platelet count gradually normalized. The patient was Child-Pugh class A during the whole follow-up period. The patient was monitored every 6 months with abdomen ultrasound and serum alpha-fetoprotein. The patient is currently treated with mesalazine alone and regularly attends follow-up visits at our center.

## 3. Discussion

### 3.1. Review of Literature

There are only two other reported cases of thiopurine-induced liver cirrhosis. Trabelsi et al. described the case of an ileocolonic steno-penetrating Crohn's disease patient who was diagnosed with liver cirrhosis during a surgical treatment for an intestinal stenosis. The patient had been treated with AZA therapy for four years, and no alterations of liver enzymes have been detected before [9]. In the second case, which was presented by De Boer et al., liver cirrhosis was discovered as further investigations were made for a new-onset thrombocytopenia, which arose after three years of therapy with azathioprine. In both cases, a liver biopsy was used to diagnose liver cirrhosis, so as to exclude noncirrhotic causes of portal hypertension (veno-occlusive disease, nodular regenerative hyperplasia, hepatic peliosis, etc.) [10].

### 3.2. What Is the Cause of Thiopurine-Induced Liver Damage?

Liver damage induced by AZA seems to be related to an accumulation of its methylated metabolites [11, 12]. Azathioprine is converted to 6-mercaptopurine (6-MP) by glutathione-S-transferase (GST). 6-MP is then converted to 6-thioguanine nucleotides (6-TGN) by the enzymes hypoxanthine-guanine phosphoribosyltransferase (HGPRT), inosine-5′-monophosphate dehydrogenase (IMPDH), and guanosine monophosphate synthetase (GMPS). 6-MP can also be converted to 6-methylmercaptopurine (6-MeMP) by thiopurine methyltransferase (TPMT) [13, 14]. Meijer et al. showed that the liver damage induced by thiopurine is twice as frequent in the patient with higher levels of 6-MeMP (>5700 pmol/8 × 10^8^ RBC) than patients with levels of 6-MeMP in range [11].

### 3.3. Genetic Mutations of TPMT: Is There a Predictive Test for Thiopurine-Induced Liver Damage?

Mutations of TPMT can lead to a modified ability to convert 6-MP in 6-MeMP. Thus, patients with modified TPMT activity, who are treated with standard doses of thiopurine (AZA 2-2, 5 mg/kg/day and 6-MP 1-1, 5 mg/kg/day), have higher levels of 6-MeMP and therefore have an increased risk of thiopurine-induced toxicity [15]. Screening for variants in TPMT may allow the identification of patients with a high risk of thiopurine-induced toxicity, to whom drugs should be administered in reduced doses [16].

## 4. Conclusions

Liver cirrhosis is a rare but possible adverse event of AZA therapy for Crohn's disease; hence, we suggest adding the routine platelet count and upper abdomen US in Crohn's disease patients who receive thiopurine treatment. If thrombocytopenia arises during AZA therapy, further investigations should be performed. Furthermore, for preventive purposes, screening for variants in TPMT should be carried out in all patients before initiating thiopurine therapy, in order to adjust the dose of the drug, reduce the risk of complications, and avoid wasting a valid therapeutic option.

## References

[1] Gomollón F., Dignass A., Annese V. (2017). 3rd European evidence-based consensus on the diagnosis and management of crohn’s disease 2016: part 1: diagnosis and medical management. *Journal of Crohn’s and Colitis*.

[2] Huang S.-Z., Liu Z.-C., Liao W.-X. (2019). Risk of skin cancers in thiopurines-treated and thiopurines-untreated patients with inflammatory bowel disease: a systematic review and meta-analysis. *Journal of Gastroenterology and Hepatology*.

[3] Hanauer S. B., Sandborn W. J., Lichtenstein G. R. (2019). Evolving considerations for thiopurine therapy for inflammatory bowel diseases-a clinical practice update: commentary. *Gastroenterology*.

[4] Ariyaratnam J., Subramanian V. (2014). Association between thiopurine use and nonmelanoma skin cancers in patients with inflammatory bowel disease: a meta-analysis. *American Journal of Gastroenterology*.

[5] Kotlyar D. S., Lewis J. D., Beaugerie L. (2015). Risk of lymphoma in patients with inflammatory bowel disease treated with azathioprine and 6-mercaptopurine: a meta-analysis. *Clinical Gastroenterology and Hepatology*.

[6] Björnsson E. S., Gu J., Kleiner D. E., Chalasani N., Hayashi P. H., Hoofnagle J. H. (2017). Azathioprine and 6-mercaptopurine-induced liver injury. *Journal of Clinical Gastroenterology*.

[7] Gearry R. B., Barclay M. L., Burt M. J., Collett J. A., Chapman B. A. (2004). Thiopurine drug adverse effects in a population of New Zealand patients with inflammatory bowel disease. *Pharmacoepidemiology and Drug Safety*.

[8] Suárez Ferrer C., Llop Herrera E., Calvo Moya M. (2016). Idiopathic portal hypertension regarding thiopurine treatment in patients with inflammatory bowel disease. *Revista espanola de enfermedades digestivas: organo oficial de la Sociedad Espanola de Patologia Digestiva*.

[9] Trabelsi A. B. E. S., Hamami E., Souguir A. (2014). Suspected azathioprine induced liver cirrhosis: an unusual side effect. *Pan African Medical Journal*.

[10] de Boer N. K. H., Mulder C. J. J., van Bodegraven A. A. (2005). Myelotoxicity and hepatotoxicity during azathioprine therapy. *The Netherlands Journal of Medicine*.

[11] Meijer B., Wilhelm A. J., Mulder C. J. J., Bouma G., van Bodegraven A. A., de Boer N. K. H. (2017). Pharmacology of thiopurine therapy in inflammatory bowel disease and complete blood cell count outcomes. *Therapeutic Drug Monitoring*.

[12] Kiefer K., El-Matary W. (2009). 6-Mercaptopurine as an alternative to azathioprine in azathioprine-induced hepatoxicity. *Inflammatory Bowel Diseases*.

[13] Van Asseldonk D., de Boer N., Peters G., Veldkamp A., Mulder C., Van Bodegraven A. (2009). On therapeutic drug monitoring of thiopurines in inflammatory bowel disease; pharmacology, pharmacogenomics, drug intolerance and clinical relevance. *Current Drug Metabolism*.

[14] Chang J. Y., Cheon J. H. (2019). Thiopurine therapy in patients with inflammatory bowel disease: a Focus on Metabolism and Pharmacogenetics. *Digestive Diseases and Sciences*.

[15] Asadov C., Aliyeva G., Mustafayeva K. (2017). Thiopurine S-methyltransferase as a pharmacogenetic biomarker: significance of testing and review of major methods. *Cardiovascular & Hematological Agents in Medicinal Chemistry*.

[16] Ford L. T., Berg J. D. (2010). Thiopurine S-methyltransferase (TPMT) assessment prior to starting thiopurine drug treatment; a pharmacogenomic test whose time has come. *Journal of Clinical Pathology*.

